# High-Sensitivity, High-Resolution Miniaturized Spectrometers for Ultraviolet to Near-Infrared Using Guided-Mode Resonance Filters

**DOI:** 10.3390/molecules29235580

**Published:** 2024-11-26

**Authors:** Jingjun Wu, Cong Wei, Hanxiao Cui, Fujia Chen, Kang Hu, Ang Li, Shilong Pan, Yihao Yang, Jun Ma, Zongyin Yang, Wanguo Zheng, Rihong Zhu

**Affiliations:** 1 School of Electronic and Optical Engineering, Nanjing University of Science and Technology, Nanjing 210094, China; jingjunwu163@163.com (J.W.); weicong@njust.edu.cn (C.W.); group_ye@163.com (W.Z.); zhurihong@njust.edu.cn (R.Z.); 2 School of Aeronautics and Astronautics, Sichuan University, Chengdu 610065, China; hanxiao.cui@scu.edu.cn (H.C.); hukang1@stu.scu.edu.cn (K.H.); 3 College of Information Science and Electronic Engineering, Zhejiang University, Hangzhou 310027, China; fujiachen@zju.edu.cn (F.C.); yangyihao@zju.edu.cn (Y.Y.); 4 Key Laboratory of Radar Imaging and Microwave Photonics, Ministry of Education, Nanjing University of Aeronautics and Astronautics, Nanjing 210016, China; ang.li@nuaa.edu.cn (A.L.); pans@nuaa.edu.cn (S.P.)

**Keywords:** miniaturized spectrometers, high sensitivity, high resolution, spectral analysis, guided-mode resonance

## Abstract

Miniaturized spectrometers have significantly advanced real-time analytical capabilities in fields such as environmental monitoring, healthcare diagnostics, and industrial quality control by enabling precise on-site spectral analysis. However, achieving high sensitivity and spectral resolution within compact devices remains a significant challenge, particularly when detecting low-concentration analytes or subtle spectral variations critical for chemical and molecular analysis. This study introduces an innovative approach employing guided-mode resonance filters (GMRFs) to address these limitations. Functioning similarly to notch filters, GMRFs selectively block specific spectral bands while allowing others to pass, maximizing energy extraction from incident light and enhancing spectral encoding. Our design incorporates narrow band-stop filters, which are essential for accurate spectrum reconstruction, resulting in improved resolution and sensitivity. Our spectrometer delivers a spectral resolution of 0.8 nm over a range of 370–810 nm. It achieves sensitivity values that are more than ten times greater than those of conventional grating spectrometers during fluorescence spectroscopy of mouse jejunum. This enhanced sensitivity and resolution are particularly beneficial for chemical and biological applications, facilitating the detection of trace analytes in complex matrices. Furthermore, the spectrometer’s compatibility with complementary metal oxide semiconductor (CMOS) technology enables scalable and cost-effective production, fostering broader adoption in chemical analysis, materials science, and biomedical research. This study underscores the transformative potential of the GMRF-based spectrometer as an innovative tool for advancing chemical and interdisciplinary analytical applications.

## 1. Introduction

Miniaturized spectrometers have emerged as crucial tools in various fields, including environmental monitoring, medical diagnostics, and industrial applications [1]. Their compact size allows for easy transport and use in field conditions, making them ideal for on-site analysis where traditional laboratory facilities are unavailable [2,3,4]. Recent breakthroughs in nanotechnology, microfabrication, and optics have enabled substantial progress in the development of miniaturized spectrometers, expanding their applications and improving their accessibility, as well as enabling on-site analysis that was previously only possible in well-equipped laboratories [5,6,7].

Despite these advances, a significant challenge persists: achieving high sensitivity and high spectral resolution simultaneously within the compact framework of miniaturized spectrometers [8,9,10]. These performance metrics are critical for the detailed analysis of complex samples, particularly where detecting low-concentration analytes is necessary [11]. However, this requirement presents substantial technical obstacles, primarily due to the inherent physical and optical limitations associated with reducing the size of spectroscopic components. High-resolution measurements typically necessitate a longer optical path or more prominent dispersive elements to spread out the spectrum sufficiently, which inherently conflicts with the objectives of miniaturization [12]. Balancing these competing demands remains a formidable challenge in the field of spectroscopy, driving ongoing research and innovation in the design of miniaturized spectrometric systems.

A promising approach involves using advanced materials and structures, such as guided-mode resonance (GMR) metasurfaces, to control light at the nanoscale [13,14]. GMR is a phenomenon in which light resonates strongly within a periodic optical structure due to phase matching between the specific wavelength of incident light and the guided mode inside the structure. GMR offers several advantages, including high diffraction efficiency (theoretically close to 100%), ultra-narrow linewidth (potentially as narrow as 0.1 nm) [15,16,17], a simple structure (where narrowband filtering can be achieved with just two to three layers), minimal sidebands (with sideband values outside the primary resonance peak effectively suppressed), and tunable parameters (allowing for precise control over resonance wavelength, linewidth, and efficiency by adjusting the geometric structure) [18,19,20,21,22]. As a result, GMR is an excellent candidate for developing high-efficiency, high-resolution, and highly integrated filters [22,23,24,25].

In this paper, we present a novel integration of GMRF devices with CMOS technology to develop a miniaturized spectrometer. This integration enhances the spectrometer’s sensitivity and resolution capabilities by employing a sophisticated mathematical algorithm that interprets coded spectral data derived from the GMRFs. The GMRFs are engineered to possess a specific transmission profile, functioning similarly to notch filters, which selectively block specific spectral bands while permitting others to pass. This selective blocking is crucial for boosting the spectrometer’s sensitivity, enabling it to detect subtle spectral variations. The narrow configuration of the band-stop features is pivotal in this design. It not only ensures high-resolution capabilities by providing fine spectral coding necessary for precise spectrum reconstruction but also maximizes energy extraction from incident light, further amplifying the instrument’s sensitivity. This approach contrasts sharply with conventional spectrometers that use band-pass filters, which typically achieve higher resolution at the expense of reduced light intensity reaching the detectors.

Additionally, the GMRF device is fabricated on a CMOS-compatible single-layer silicon nitride (Si_3_N_4_) film, facilitating cost-effective mass production. Furthermore, by implementing a polarization-insensitive design in the GMRFs, we significantly increase the luminous flux of the spectrometer, thereby further improving its sensitivity. By optimizing both high sensitivity and high resolution, our spectrometer offers the ability to resolve fine spectral details, positioning it as an indispensable tool for chemical analysis, materials science, and biomedical research.

## 2. Results and Discussion

### 2.1. Design of GMRF Device

**Figure 1:**
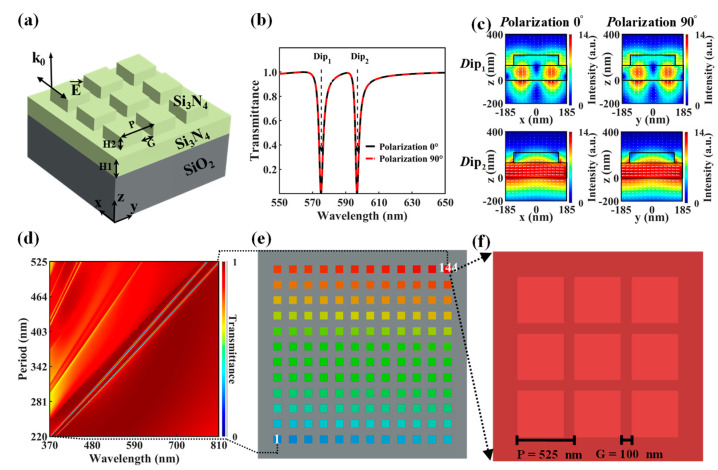
The GMRF device design. (**a**) Diagram illustrating the GMRF design. (**b**) Transmittance of nanopillar resonators with spectrally separate dips (*H*1 = 130 nm, *H*2 = 90 nm, *P* = 370 nm, and *G* = 100 nm) under 0- and 90-degree polarization. (**c**) Planar field vector plots present in nanopillar resonators at Dip1 573.4 nm and Dip2 594.9 nm. (**d**) Simulated transmittance spectra of GMRF device with 144 GMRFs. (**e**) Construction of the GMRF device. (**f**) Partially enlarged details of the GMRF are marked by the black box in (**e**).

The design of our GMRF device is shown in Figure 1. According to the below device design, a Si_3_N_4_ slab and a Si_3_N_4_ grating were selected to design a GMRF, as demonstrated in Figure 1a. The GMRF consists of an array of nanopillar resonators organized in a square grid. These resonators are spaced with a period (*P*) and have a fixed gap (*G*) of 100 nm. This equal spacing between nanopillars avoids the aspect ratio-dependent etching loading effect and ensures the processing accuracy of the device [26,27]. The nanopillars are created by etching a layer of Si_3_N_4_ that is 220 nm thick and placed on a quartz substrate. The nanopillars have a height (H2) of 90 nm and a waveguide layer that is 130 nm in thickness. Si_3_N_4_ is selected for its low absorption and high index contrast with the quartz substrate (Figure S1 in the Supplementary Materials). This method of preparing a GMRF device using a single layer and a single patterning step simplifies the processing steps and ensures the device’s stability [28,29]. Additionally, Si_3_N_4_ is fully compatible with standard CMOS technology, enabling cost-effective mass production [30,31].

Figure 1b displays the transmittance of the GMRF, which has a period (*P*) of 370 nm and a gap (*G*) of 100 nm for both x- and y-polarizations. This demonstrates that the GMRF is not affected by polarization. Two distinct transmittance dips occur at wavelengths of 573.4 nm and 594.9 nm. The electric field pattern distribution of the two dips under x- and y-polarizations further demonstrates their polarization insensitivity, as shown in Figure 1c. The GMRF ensures equal sensitivity to light regardless of polarization, as indicated by the 0°–90° polarization simulation results shown in Figure S2 in the Supplementary Materials. This design prevents the need for a polarizer for filtering input light, hence augmenting the photon throughput to the detector array [32,33]. By manipulating the period of the nanopillars, it is possible to regulate the resonance wavelength. As shown in Figure 1d, increasing the nanopillar period from 220 nm to 525 nm extends the resonant wavelength range from 370 nm to 810 nm.

The GMRF device consists of an array of 12 × 12 GMRFs. The nanopillar resonators are scaled linearly, with periods ranging from *P* = 220 nm (G = 100 nm) to *P* = 525 nm (*G* = 100 nm), as shown in Figure 1e,f. These gaps and periodicities match the values in the numerical simulations shown in Figure 1d. The GMRF device was fabricated using electron beam lithography (EBL), with detailed processing procedures provided in Figure S3 in the Supplementary Materials.

### 2.2. Fabrication and Characterization of GMRF Device

**Figure 2:**
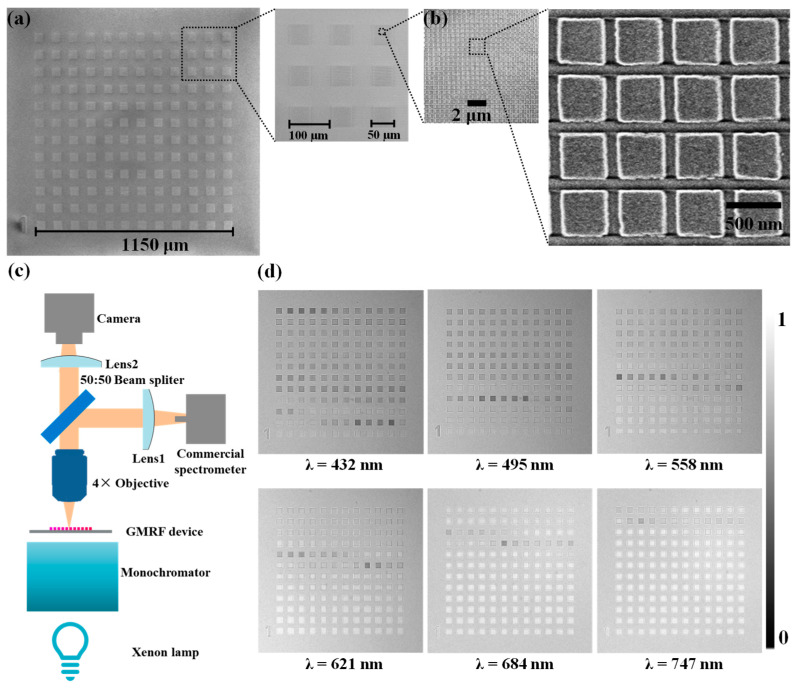
The calibration of the GMRF device. (**a**,**b**) SEM image of the GMRF device. (**c**) Homemade spectral filter detection setup. (**d**) The filtering effect of the GMRF device under different wavelengths.

Scanning electron microscopy (SEM) analyses confirmed the precision of the GMRF device fabrication. Figure 2a,b demonstrate the consistent scaling of the nanopillar resonator geometries across the entire array of GMRF devices. The optical transmittance properties were evaluated using a homemade setup, as shown in Figure 2c. This setup offered high-resolution microscopy capabilities, equipped with a 2048 × 1024 pixel array imaging detector (Indigo Inc., model S200MRU, Fuzhou, China) and a spectrum of linearly variable light sources spanning the visible range. A 4× refractive objective was used to capture the optical responses from all GMRF devices. A 50:50 beam splitter (BS) was employed to obtain the spectrum while simultaneously capturing a clear image. Transmittance images for different wavelengths of the incident visible light, with image intensities normalized, are presented in Figure 2d, showcasing the device’s excellent spectral encoding properties.

**Figure 3:**
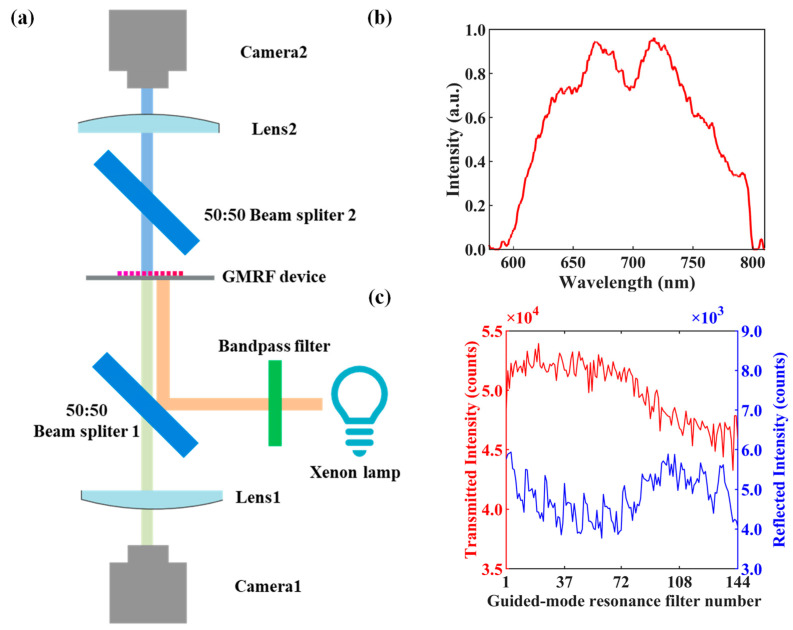
Luminous flux comparison between transmitted and reflected light. (**a**) Test setup schematic of luminous flux comparison of the transmitted band-stop and reflected band-pass light. (**b**) The incident test spectrum. (**c**) Averaging intensity comparison between the transmitted and reflected light.

We created a test configuration to assess the luminous flux of the band-pass and band-stop filtering of the GMRFs, as shown in Figure 3a. Broadband light emitted from a xenon lamp is directed onto the GMRF device, with a band-pass filter removing light outside the test band. Camera 1 detects the reflected light, while Camera 2, an identical camera, measures the transmitted light. Due to the 50:50 beam splitter 1, the reflected light loses 50% of its energy. Another 50:50 beam splitter 2 is placed in the transmission path to compensate for this loss. To accurately compare the sensitivity of the band-pass and band-stop filters, it is essential to ensure that the image size of the filters is identical on both cameras and that the numerical aperture of the camera diaphragm is the same. Figure 3b shows the testing setup for the 144 GMRF device under broadband illumination. Figure 3c presents the average transmitted and reflected intensities for the 144 GMRFs. The results indicate that the transmitted band-stop intensities are more than seven times greater than the reflected band-pass intensities.

### 2.3. Integration and Performance Testing of Our Spectrometer

**Figure 4:**
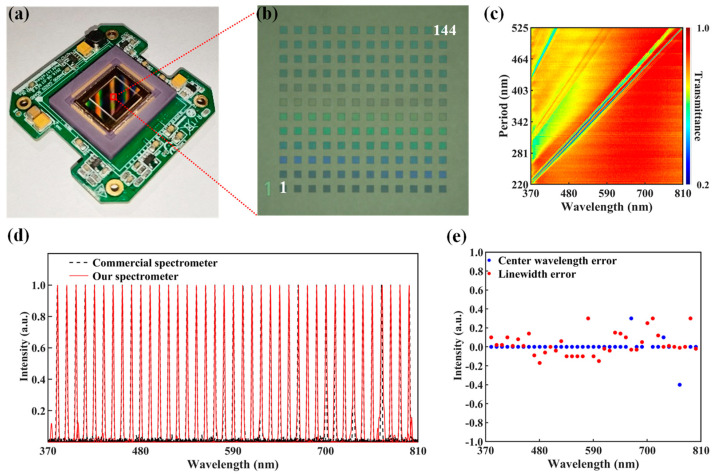
GMRF device-based micro-spectrometer. (**a**) Diagram showing the spectrometer mounted atop a CMOS image sensor and equipped with a GMRF device. (**b**) Microscope image of the GMRF device, the number 1 corresponds to a period of 220 nm, while the number 144 corresponds to a period of 525 nm. (**c**) Band-stop *T*_i_(λ) transmittance profiles for the spectrometer as a whole. (**d**) Visible light spectrum narrowband measurements were obtained using both a commercial spectrometer and our spectrometer. (**e**) The reconstruction errors of the central wavelength and linewidth for the results in (**d**).

We developed a high-performance micro-spectrometer utilizing our GMRF device. Instead of a direct readout, the spectrometer processes the light signal using an array of GMRFs arranged on a CMOS image sensor, as illustrated in Figure 4a,b. The spectra are then computationally reconstructed. Figure 4c displays the transmittance characteristics *T_i_*(*λ*) of our spectrometer, measured with a modified micro-area UV-VIS spectrophotometer. A schematic diagram of the apparatus used for calibrating the spectral response of the spectrometer is illustrated in Figure S4. The multilayer band-stop feature at shorter wavelengths does not affect the spectrometer’s performance, as it is accounted for during system calibration. The system’s spectral response profile, *T_i_*(*λ*), is the multiplication of the dispersion curve of the lens imaging system, the GMRF array’s transmittance profiles, and the CMOS pixels’ photoresponse profiles.

We compared the device’s performance with a commercial spectrometer to demonstrate its accuracy. We verified the accuracy of our spectrometer’s reconstruction by assessing a sequence of narrow spectral lines within the 370–810 nm range. The schematic diagram of the setup used for testing the accuracy of the spectrometer is identical to the one used for calibration, as shown in Figure S4 in the Supplementary Materials. Figure 4d demonstrates a tight consistency between the results obtained from our spectrometer and those acquired from commercially available spectrometers (Avaspec-uls2048cl, Avantes, Apeldoorn, The Netherlands). Most reconstruction errors for the center wavelength were ±0.1 nm, with only rare deviations, as shown in Figure 4e. The deviation of the reconstruction errors for the linewidth also achieved a very high accuracy of ±0.3 nm.

**Figure 5:**
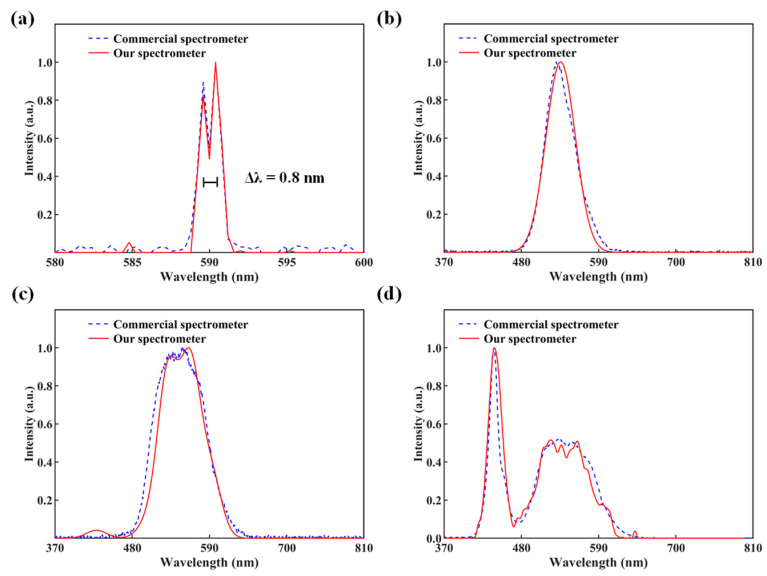
Spectrum measurements were obtained using both a commercial spectrometer and our spectrometer. (**a**) Both the commercial spectrometer and our spectrometer capture the spectrum of two narrow spectral lines. (**b**–**d**) show broad-spectrum measurements.

The spectral resolution of our spectrometer was assessed using the Rayleigh criterion, which stipulates that two spectral lines are considered resolved when the highest point of the first peak aligns with the lowest point of the second peak. As shown in Figure 5a, our spectrometer achieved a spectral resolution of 0.8 nm. For a more comprehensive view, the complete results for the 370–810 nm range are provided in Figure S5 in the Supplementary Materials. Figure 5b–d display broad spectra of various morphologies, demonstrating that our spectrometer can accurately reconstruct continuous broadband spectra across the operational wavelength span. The schematic diagram of the setup used to test the spectral resolution and broadband spectral performance of the spectrometer is the same as the one used for calibration, with the only difference being the light source.

**Figure 6:**
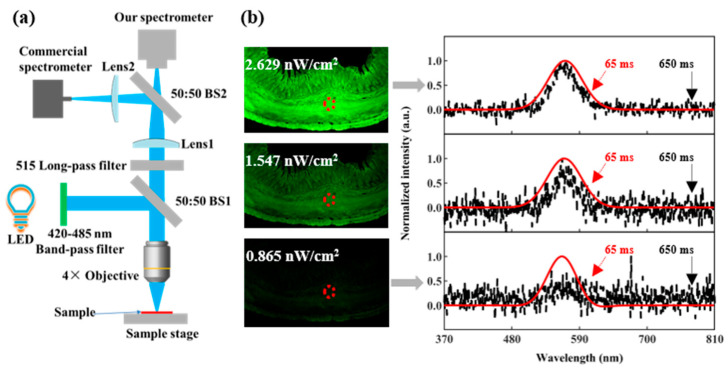
Weak light detection testing of our spectrometer. (**a**) Diagram of the experimental setup used for low-light fluorescence measurement of the mouse jejunum. (**b**) Fluorescence images (the left images) and the spectra of a commercial spectrometer (black) and our spectrometer (red) under varying levels of illumination, the red circle indicates the fluorescence spectrum measurement region.

A vital feature of this platform is its exceptional sensitivity, which allows it to maximize the limited light available in compact devices. Our spectrometer’s spectroscopic sensitivity may be demonstrated using a sample of mouse jejunum for local fluorescence measurements. Figure 6a depicts the test configuration used for this investigation. A band-pass filter ranging from 420 to 485 nm is used to select the fluorescence excitation from the broadband light emitted by the LED source. Via a 4× microscope lens, light is reflected onto a fluorescent sample via a 50:50 beam splitter (BS1). Excitation light that is reflected, and fluorescence is collected by the same objective. The only light left over after excitation is the fluorescence signal, as all other signals have been removed by the 515 nm long-pass filter. The fluorescence is divided equally by a 50:50 beam splitter (BS2), with one beam incident on our spectrometer and the other beam entering the commercial spectrometer.

In Figure 6b, the left side displays fluorescence images of the mouse jejunum (provided by Henan Dake Educational Instruments Co., Ltd., Zhengzhou, China) under varying illumination levels (the complete picture of the mouse jejunum is provided in Figure S6 in the Supplementary Materials). The right side presents the test results of the fluorescence spectrum obtained using a commercial spectrometer (black) and our spectrometer (red). As the power diminishes, the spectra obtained from the commercial spectrometer are inundated with noise, whereas our spectrometer can still precisely retrieve the spectrum. Notably, the integration time of our spectrometer is one-tenth of that of the commercial spectrometer. This proves that the sensitivity of our spectrometer is more than 10 times that of commercial spectrometers. When evaluating biological samples, enabling low-light excitation fluorescence measurements might assist in preventing damage or photobleaching. As a power meter cannot measure the irradiance, we approximate the provided values by utilizing the spectrometer’s linear power response (Figure S7 in the Supplementary Materials) with an exposure duration of 65 ms. This linear relationship indicates that the fluorescent signal’s power densities are 2.629 nW/cm^2^, 1.547 nW/cm^2^, and 0.865 nW/cm^2^, respectively.

## 3. Materials and Methods

### 3.1. Device Design

The GMRF device design is based on the principle of GMR. Generally, GMR is formed by combining a subwavelength periodic diffraction grating with a slab waveguide [28,34,35]. Therefore, there are two physical mechanisms. The first is grating diffraction. The magnitudes of the diffracted modes are determined by solving Maxwell’s equations, while the orientations are quantified using the well-known grating equation [36,37]. The second mechanism is slab guiding. Wave guiding is only possible if the effective dielectric constant of the guided mode is higher than that of the surrounding media and lower than the dielectric constant of the slab. GMR occurs only when the angle of the diffracted mode precisely matches that of a guided mode in the slab [38,39]. When GMR occurs, a narrow resonant feature emerges in the reflection or transmission spectrum [29,40]. Therefore, the subwavelength grating and waveguide properties of the GMR structure can be designed to achieve the desired spectral selectivity. In the non-resonant wavelength region, the transmission and reflection spectra can be understood using conventional thin-film theory [41].

The GMRFs were designed and simulated using the finite-difference time-domain approach. Enclosed, the nanopillar resonator simulation region had periodic boundary conditions on the x- and y-axes and perfectly matched layers on the z-axis. A plane wave with x-polarization shone light on the structure. Optical constants used in the simulation were derived from experimental data (Figure S1 in the Supplementary Materials). To optimize the performance of the GMRFs, we systematically varied the geometric parameters, including the period (*P*) and the heights (H1 and H2). The simulation was configured with a mesh accuracy setting of 8 (corresponding to 10.88 nm), and an additional finer mesh with a resolution of 5 nm was applied in the z-direction.

### 3.2. Device Fabrication

The fabrication of our GMRF device involved two primary processes: depositing a Si_3_N_4_ layer and forming nanopillars within this film. We deposited the Si_3_N_4_ film onto a quartz substrate using electron beam evaporation (Heng Yue vacuum SP800, Sanhe Hengyue Vacuum Equipment Co., Ltd., Langfang, China). Before deposition, the substrates underwent a series of 20 min ultrasonic cleaning cycles in acetone, methanol, and deionized water. The deposition rate was 0.8 Å/s, and the electron beam evaporation was performed in a base vacuum with a pressure of 2 × 10^−7^ Torr. As soon as the Si_3_N_4_ films were deposited, ellipsometry was used to quantify their refractive index (*n*) and absorption coefficient (*k*). The results showed a refractive index of approximately 1.9 and no absorption within the 370–810 nm wavelength range (Figure S1 in the Supplementary Materials).

The next step was to spin-coat the device with a 300 nm ARP layer and bake it at 85 °C for 90 min. We used an electron beam writer (Elionix ELS-F125, Elionix Inc., Tokyo, Japan) with an acceleration voltage of 125 kV to create nanopillar lattice patterns in the ARP. The ARP with a pattern was created using AR600.546 and deionized water at a temperature of 25 °C for durations of 60 and 30 s, respectively. Afterward, a 50 nm layer of chromium (Cr) was deposited using an electron beam evaporator (ULVAC EI-501z, ULVAC, Kanagawa, Japan), and the mask was removed using N-methyl-2-pyrrolidone (NMP) at a temperature of 120 °C for roughly two hours. The Si_3_N_4_ layer was then etched using the Inductively Coupled Plasma (ICP) method (Leuven Instruments, Xuzhou, China). Finally, a wet chemical etch was employed to remove the Cr mask layers.

### 3.3. Principle of Computational Spectrometer

The spectral information captured by our spectrometer can be reconstructed using a computational strategy. The incident light illuminates the GMRFs via the lens imaging system, and the transmitted light power from each filter is measured by a photodetector. The incident light spectra can be reconstructed by using the measured power and known pre-calibrated response functions *T_i_*(*λ*), the reconstruction algorithm partially refers to the algorithms of a nanowire spectrometer [42]. The core technique for detecting spectra involves filtering the spectra into intensities:

(1) $$\int_{\lambda_{\min}}^{\lambda_{\max}}S(\lambda )T_{i}(\lambda )d\lambda =I_{i}\;(i=1,2,3,\cdots m)$$

where *m* is the number of detectors. As shown in Equation (1), the unknown incident spectrum *S*(*λ*) can be reconstructed by solving equations composed of signal intensities *I_i_* and pre-calibrated response functions *T_i_*(*λ*). The *T_i_*(*λ*) is the multiplication of the dispersion curve of the lens imaging system, the transmission response of the GMRF, and the absorption quantum efficiency of the CMOS image sensor. Where the integral equations are discretized to produce a matrix equation, as shown in Equation (2).

(2) $$\left[\begin{array}{cccc} T_{11} & T_{12} & \cdots & T_{1n}\\ T_{21} & T_{22} & \cdots & T_{2n}\\ \vdots & \vdots & \vdots & \vdots \\ T_{m1} & T_{m2} & \cdots & T_{mn} \end{array}\right]\left[\begin{array}{c} S_{1}\\ S_{2}\\ \vdots \\ S_{n} \end{array}\right]=\left[\begin{array}{c} I_{1}\\ I_{2}\\ \vdots \\ I_{m} \end{array}\right]$$

Mathematically, each detector’s incidence light intensity (*I_i_*) may be described by a group of linear equations. These equations are obtained by multiplying the incident spectrum *S*(*λ*) with the *T_i_*(*λ*) transmission profile. A reconstruction approach (Part S2 in the Supplementary Materials) solves the equations collected by all detectors, allowing us to approximate *S*(*λ*). Unlike previous works that typically use band-pass or random-pass filter functions, we propose a narrow band-stop filtering method derived from GMRFs to improve luminous flux and, consequently, the spectrometer’s sensitivity.

## 4. Conclusions

In conclusion, we have demonstrated that engineering GMR offers a novel approach to micro-spectrometers, combining high resolution with sensitivity. Firstly, the GMRF device was carefully designed and constructed. We subsequently developed a spectrometer that operates within the wavelength range of 370–810 nm, achieving a resolution of 0.8 nm by integrating the GMRF device with a CMOS camera. Mouse jejunum fluorescence spectrum tests demonstrate that the sensitivity of our spectrometer exceeds that of traditional grating spectrometers by more than tenfold. This design represents a significant breakthrough in spectroscopy, offering enhanced performance, reduced device size, and a versatile solution that seamlessly integrates with existing semiconductor technologies. Consequently, it holds substantial potential for a wide range of low-light applications, including chemical and nanoscale biological spectroscopy, astronomical spectroscopy, and Raman research.

## Data Availability

The data that support the plots within this paper are available from the corresponding authors upon request.

## Supplementary Materials

The following supporting information can be downloaded at https://www.mdpi.com/article/10.3390/molecules29235580/s1. Figure S1: Optical properties of the Si_3_N_4_ film. Showing refractive index, *n* (red), and absorptive coefficient, *k* (blue); Figure S2: Transmittance of nanopillar resonators with spectrally separate dips (*H*1 = 130 nm, *H*2 = 90 nm, *P* = 370 nm, *G* = 100 nm) under 0°—90° polarization. Figure S3: Schematic showing the fabrication process of a nanopillar lattice in the Si_3_N_4_ film to create the GMRF device. Figure S4: Schematic diagram of the apparatus for calibrating the spectral response of our spectrometer. Figure S5: Spectrum measurements were obtained using both our spectrometer and a commercial spectrometer to analyze two narrow spectral lines. Figure S6: Mouse jejunum samples. (a) Overall morphology of the mouse jejunum sample. (b) Partial fluorescence photographs of the mouse jejunum sample under different excitation intensities. Figure S7: Readout intensities of the CMOS chip as a function of the incident power density with an exposure time of 65 ms.
