# Supplementary material for: High-Sensitivity, High-Resolution Miniaturized Spectrometers for Ultraviolet to Near-Infrared Using Guided-Mode Resonance Filters
Source: Molecules. 2024 Nov 26;29(23):5580. doi: 10.3390/molecules29235580 (PMC11643753; doi:10.3390/molecules29235580)
Supplement: Supplementary file 1 [file molecules-29-05580-s001.zip › molecules-3308525-supplementary.pdf]

High-sensitivity, high-resolution miniaturized spectrometers for ultraviolet to near-infrared using guided-mode resonance filters

Jingjun Wu [1,†], Cong Wei [1,†], Hanxiao Cui [3], Fujia Chen [2], Kang Hu [3], Ang Li [4], Shilong Pan [4], Yihao Yang [2], Jun Ma [1,*], Zongyin Yang [2,*], Wanguo Zheng [1] and Rihong Zhu [1]

[1] School of Electronic and Optical Engineering, Nanjing University of Science and Technology, Nanjing, 210094, China.
[2] College of Information Science and Electronic Engineering, Zhejiang University, Hangzhou, 310027, China.
[3] School of Aeronautics and Astronautics, Sichuan University, Chengdu, 610065, China.
[4] Key Laboratory of Radar Imaging and Microwave Photonics, Ministry of Education, Nanjing University of Aeronautics and Astronautics, Nanjing, 210016, China.
* Correspondence: * majun@njust.edu.cn, yangzongyin@zju.edu.cn.
† These authors contributed equally to this work.

Part S1: extended data figures

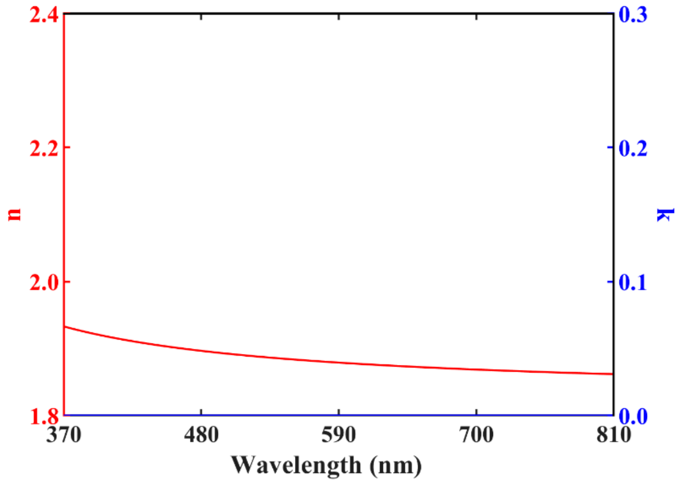

Figure S1. Optical properties of the Si_3N_4 film. Showing refractive index, *n* (red), and absorptive coefficient, *k* (blue).

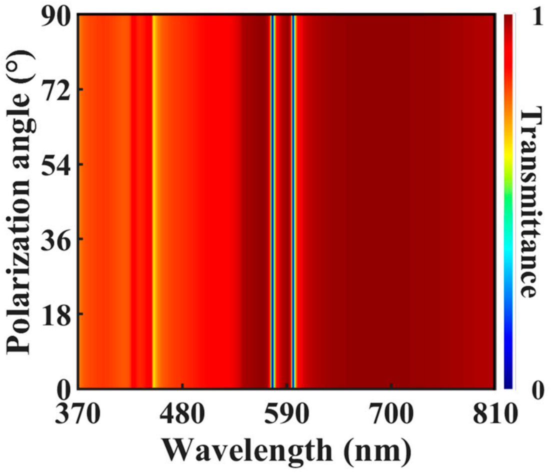

Figure S2. Transmittance of nanopillar resonators with spectrally separate dips (H1 = 130 nm, H2 = 90 nm, P = 370 nm, G = 100 nm) under 0º – 90º polarization.

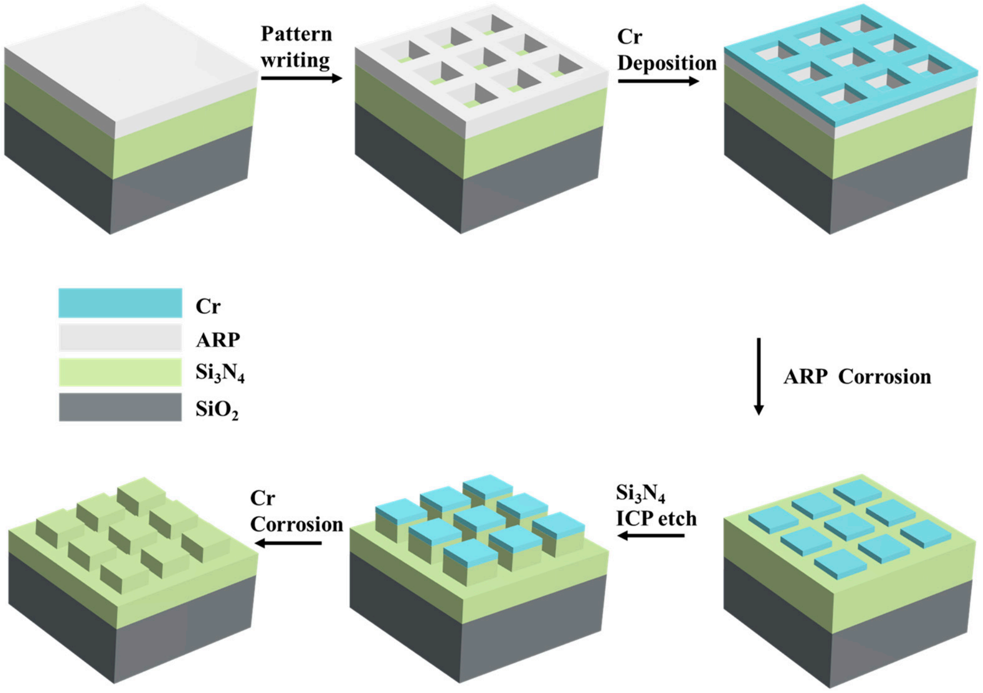

Figure S3. Schematic showing the fabrication process of a nanopillar lattice in the Si_3N_4 film to create the GMRF device.

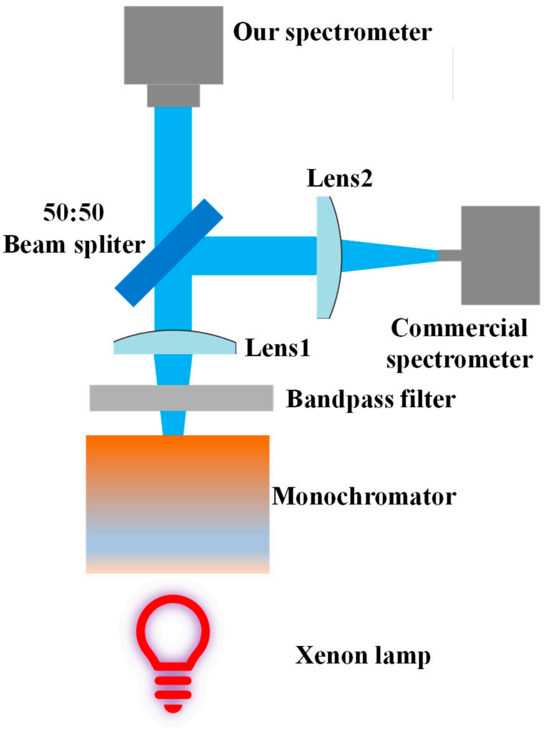

Figure S4. Schematic diagram of the apparatus for calibrating the spectral response of our spectrometer.

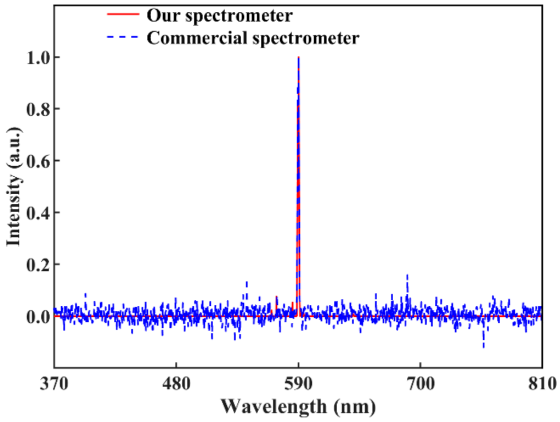

Figure S5. Spectrum measurements were obtained using both our spectrometer and a commercial spectrometer to analyze two narrow spectral lines.

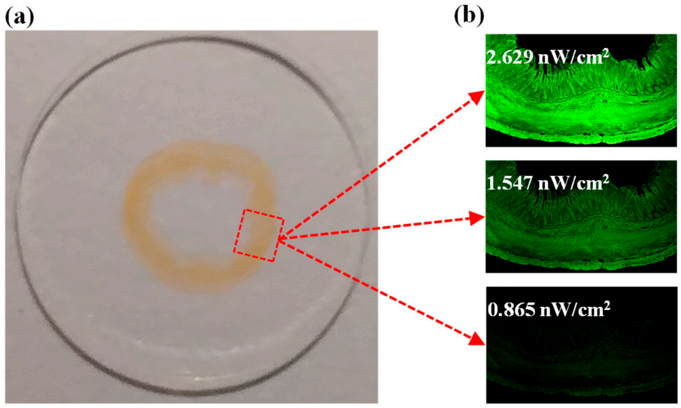

Figure S6. Mouse jejunum samples. (a) Overall morphology of the mouse jejunum sample. (b) Partial fluorescence photographs of the mouse jejunum sample under different excitation intensities.

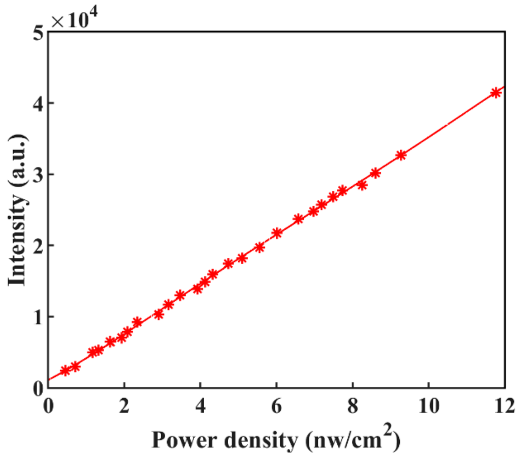

Figure S7. Readout intensities of the CMOS chip as a function of the incident power density with an exposure time of 65 ms.

Part S2: Reconstructive algorithms

Our spectral reconstruction algorithm partially refers to the algorithms of a nanowire spectrometer [1], in which a linear combination of Gaussian basis functions with different amplitudes is exploited to fit the target spectrum. The details are shown below:

The photoelectric signals I_i captured on the CMOS imaging sensor can be expressed as the integral of the incident spectrum $S(\lambda)$ and the spectral response functions (equals the product of the transmission and the quantum efficiency of CMOS sensors) of meta-pixels $T_i(\lambda)$:

$$\int_{\lambda_{min}}^{\lambda_{max}} S(\lambda) T_i(\lambda) d\lambda = I_i \quad (i = 1, 2, 3, \cdots m) \tag{1}$$

Where λ_{min} and λ_{max} are the minimum and maximum wavelengths of the incident spectrum, and m is the number of meta-pixels. Since the spectral reconstruction is an ill-posed problem, the equations cannot be solved directly and require an appropriate

formal transformation. By decomposing the incident spectrum $S(\lambda)$ into a linear combination of several Gaussian basis functions $\varphi_j(\lambda)$ and the weight factors α_j, $S(\lambda)$ can be expressed as:

$$S(\lambda) \approx \sum_{j=1}^{n} \alpha_j \varphi_j(\lambda) \tag{2}$$

where $\varphi_j(\lambda)$ is a Gaussian function with a peak at λ_j, and it can be expressed as:

$$\phi_j(\lambda) = \frac{1}{\sigma\sqrt{2\pi}} exp\left[-\frac{1}{2}\left(\frac{\lambda-\lambda_j}{\sigma}\right)^2\right] \tag{3}$$

Where σ is the width of the Gaussian function. Considering Equation 2 and Equation 3 and Equation 1, we get:

$$\sum_{j=1}^{n}\left(\int_{\lambda_{min}}^{\lambda_{max}} T_i(\lambda)\varphi_j(\lambda)d\lambda\right)\alpha_j = I_i \tag{4}$$

The equation above can be expressed as:

$$A\alpha = c \tag{5}$$

Where A is a matrix with size $m{\times}n$ with the element $a_{ji} = \int R_i(\lambda)\varphi_j(\lambda)d\lambda$ $c = [I_1, I_2, \cdots, I_m]^T$, $\alpha = [\alpha_1, \alpha_2, \cdots, \alpha_n]^T$, it is the unknown vector. Solving a linear system of equations can be transformed into a minimum residual problem, i.e., minimizing the residual norm $e = \|Aa - c\|_2$.

Because the residual minimization of these linear equations is sensitive to high-frequency noise, it can easily lead to numerical instability. A suitable regularization process is required to solve the instability problem. In addition, the quality of the reconstructed spectra is also related to the choice of the σ-width of the Gaussian basis function. In our study, the nonnegative linear least-square method is exploited.

Part S3: Calibration

A custom-built calibration setup was developed to calibrate the spectral characteristics of the proposed spectrometer. As shown in Figure S4, a Xenon lamp (Microsolar 300) was used as the light source, providing white light. This light was spatially dispersed by a monochromator (CME-Mo301), allowing for the selection of monochromatic light by adjusting the grating's position. A band-pass filter was employed to eliminate harmonic waves generated by the grating within the monochromator. The output light had a full width at half maximum (FWHM) of 0.5 nm. The calibration light was then split using a 50:50 beam splitter, directing one beam to the spectrometer chip and the other to a commercial spectrometer. The commercial spectrometer was used to monitor both the central wavelength and the power of the calibration light.

References

1. Yang, Z.; Albrow-Owen, T.; Cui, H.; Alexander-Webber, J.; Gu, F.; Wang, X.; Wu, T.-C.; Zhuge, M.; Williams, C.; Wang, P.; et al. Single-nanowire spectrometers. *Science* **2019**, *365*, 1017-1020, doi:10.1126/science.aax8814.
